# Integrating Serum Metabolome and Gut Microbiome to Evaluate the Benefits of Lauric Acid on Lipopolysaccharide- Challenged Broilers

**DOI:** 10.3389/fimmu.2021.759323

**Published:** 2021-10-15

**Authors:** Yanping Wu, Qing Li, Jinsong Liu, Yulan Liu, Yinglei Xu, Ruiqiang Zhang, Yang Yu, Yongxia Wang, Caimei Yang

**Affiliations:** ^1^ College of Animal Science and Technology, College of Veterinary Medicine, Zhejiang Agricultural and Forestry University, Hangzhou, China; ^2^ Institute of Animal Health Products, Zhejiang Vegamax Biotechnology Co., Ltd., Anji, China

**Keywords:** lauric acid, LPS challenge, broilers, serum metabolism, gut microbiota

## Abstract

Lauric acid (LA) is a crucial medium-chain fatty acid (MCFA) that has many beneficial effects on humans and animals. This study aimed to investigate the effects of LA on the intestinal barrier, immune functions, serum metabolism, and gut microbiota of broilers under lipopolysaccharide (LPS) challenge. A total of 384 one-day-old broilers were randomly divided into four groups, and fed with a basal diet, or a basal diet supplemented with 75 mg/kg antibiotic (ANT), or a basal diet supplemented with 1000 mg/kg LA. After 42 days of feeding, three groups were intraperitoneally injected with 0.5 mg/kg *Escherichia coli*- derived LPS (LPS, ANT+LPS and LA+LPS groups) for three consecutive days, and the control (CON) group was injected with the same volume of saline. Then, the birds were sacrificed. Results showed that LA pretreatment significantly alleviated the weight loss and intestinal mucosal injuries caused by LPS challenge. LA enhanced immune functions and inhibited inflammatory responses by upregulating the concentrations of immunoglobulins (IgA, IgM, and IgY), decreasing IL-6 and increasing IL-4 and IL-10. Metabolomics analysis revealed a significant difference of serum metabolites by LA pretreatment. Twenty-seven serum metabolic biomarkers were identified and mostly belong to lipids. LA also markedly modulated the pathway for sphingolipid metabolism, suggesting its ability to regulate lipid metabolism. Moreover,16S rRNA analysis showed that LA inhibited LPS-induced gut dysbiosis by altering cecal microbial composition (reducing *Escherichia-Shigella*, *Barnesiella* and *Alistipes*, and increasing *Lactobacillus* and *Bacteroides*), and modulating the production of volatile fatty acids (VFAs). Pearson’s correlation assays showed that alterations in serum metabolism and gut microbiota were strongly correlated to the immune factors; there were also strong correlations between serum metabolites and microbiota composition. The results highlight the potential of LA as a dietary supplement to combat bacterial LPS challenge in animal production and to promote food safety.

## Introduction

Medium-chain fatty acids (MCFAs) are saturated 6-12 carbon fatty acids that are abundant in milk lipids and oil fractions of various plants such as coconuts, palm kernels and cuphea seeds (1). They can be efficiently absorbed and metabolized even by newborn and suckling young, and provide many physiological benefits including metabolism modulation, immune enhancement and pathogen inhibition (2–4). In recent years, MCFAs have been gradually used as feed supplements to enhance animal growth and health, and consequently improve the quality of animal-derived food products (5). Lauric acid (LA) is a primary member of MCFAs that consists of 12 carbon atoms (C12:0). Accumulating evidence has revealed its beneficial effects on animals. LA supplementation could markedly improve the growth performance and health of weaned piglets (6). In poultry production, LA showed a strong ability to reduce the growth and colonization of pathogenic bacteria such as *Campylobacter jejuni* and *Clostridium perfringens* in chickens (7, 8). Recently, it was reported that LA can be a promising alternative strategy to antibiotics in livestock nutrition (9). The possible mechanism is that LA has broad-spectrum antibacterial, antiviral and antifungal activities (10). It can penetrate bacterial cell membranes, destabilize their structures and cause cell lysis, and moreover, bacteria are unlikely to acquire resistance (9). 

Microbial infection is considered as a significant threat to animal production and food safety. Lipopolysaccharide (LPS), a membrane glycolipid, is a key component of gram-negative bacterial cell membrane and drives diseases (11). Animals are highly sensitive to the toxic effects of LPS. It has been reported that the intraperitoneal injection of LPS results in various physiological changes. LPS can activate an acute immune response and induce systemic dysfunction by producing proinflammatory factors, restricting the expression of innate immune receptors and downregulating the synthesis of immunoglobulins (12). Antibiotic use is the main approach to confront LPS challenge; however, antibiotic overuse may lead to the growth of antibiotic-resistant pathogens. Accordingly, LA supplementation might be a potential strategy to solve this problem owing to its superior antimicrobial effects.

Bacteria challenge can lead to host metabolic changes through their cell envelope components, particularly LPS (13). LPS possesses a strong ability to reprogram metabolism by promoting aerobic glycolysis, and modifying amino acid and lipid metabolism (14–16). Many studies have demonstrated that the differential expression of metabolites is involved in the promotion and regulation of LPS-mediated inflammation (16, 17). Thus, regulating host metabolism may be an effective way to alleviate LPS-induced inflammation. LA has recently been shown to modulate metabolism. For example, Zhang et al. showed that LA alleviated the impaired metabolism and thermogenesis in female mice fed with a high-fat diet (18). LA could suppress the proliferation of mouse colon cancer cells by remodeling energy metabolism (19). Also, LA significantly reduced liver lipids in freshwater Atlantic salmon (20). However, to the best of our knowledge, no studies have investigated the effects of LA on metabolic changes following a bacterial infection. In this study, an HPLC/MS-based metabolomic analysis was performed to detect the changes of serum metabolome of broilers in a bacterial LPS-challenged model; then, 16S rRNA analysis was conducted to investigate the composition of cecal microbiota; and finally, Pearson’s correlation assays were performed to clarify the contributions of the altered serum metabolites and gut microbes in alleviating LPS-induced injuries and inflammation.

## Materials and Methods

### Animals and Experimental Design

A total of 384 one-day-old male Ross 308 broilers were randomly allocated to four groups with eight replicates for each group and 12 birds per replicate, and raised in an air-conditioned room and free to feed and water. The broiler chicks were fed with a basal diet (192 birds in two groups), or a basal diet supplemented with 75 mg/kg aureomycin (antibiotic, ANT, 96 birds), or a basal diet supplemented with 1000 mg/kg LA (96 birds).The basal diet was formulated to meet the recommended nutrient content by the National Research Council (1994) and was shown in **Table 1** (starter diet: days 1-21; finisher diet: days 22-44). ANT and LA were supplemented by mixing with premix and then mixing thoroughly with the basal diet. The dosages of ANT and LA were chosen according to our previous study (21). The feeding period was 42 days. On day 42, one bird from the eight replicates of the four groups were randomly taken for LPS challenge. The selected 32 birds were weighed and intraperitoneally injected with 0.5 mg/kg *Escherichia coli*- derived LPS (Sigma-Aldrich Inc., St. Louis, MO, USA) or the same volume of saline thrice on days 42, 43 and 44. Specifically, the four groups (N=8 in each group) were as follows: negative control group (CON), fed with a basal diet and injected with saline; LPS-challenged group (LPS), fed with a basal diet and exposed to 0.5 mg/kg LPS; ANT+LPS group, fed with a basal diet containing 75 mg/kg ANT, and challenged with 0.5 mg/kg LPS; and LA+LPS group, fed with a basal diet containing 1000 mg/kg LA, and challenged with 0.5 mg/kg LPS.

**Table 1:** Composition and nutrient levels of the basal diet (air-dry basis) %.

Items	1 to 21 days of age	22 to 44 days of age
Ingredients		
Corn	52.5	54
Soybean meal	25	17
Extruded soybean	4.5	3.5
DDGS	8.5	7.5
Rice bran		6
Corn gluten		2
Soybean oil	1.7	4.6
Limestone	1.4	1.4
Fermented soybean meal	2.4	
Premix^*^	4	4
Total	100.00	100.00
Nutrient levels		
CP	22.02	19.11
ME(MJ/kg)	12.23	12.91
Crude fat	5.5	8.6
Lys	1.18	0.97
Met	0.54	0.45
Met+Cys	0.88	0.74
Thr	0.86	0.71
Try	0.23	0.20
Ca	0.82	0.73
TP	0.65	0.57

^*^Premix, per Kg of diets: 10,000 IU of vitamin A; 2.2 mg of vitamin B1; 8.0 mg of vitamin B2; 1.3 mg of vitamin K; 10 mg of pantothenic acid; 40 mg of nicotinic acid; 0.04mg of biotin; 400 mg of choline chloride; 7.5 mg of copper; 110 mg of manganese; 80 mg of iron; 65 mg of zinc; 0.18 mg of iodine; 0.15 mg of selenium.

### Sample Collection

After LPS challenge, broilers were weighed and sacrificed by injecting 20 mg/kg sodium pentobarbital. Blood was obtained from the wing vein using procoagulation tubes, and blood serum was separated by centrifugation at 4000 g for 10 min at 4°C. The intestinal samples were dissected from the mesenteric tissues. Approximately, 2 cm segments of the jejunum and ileum were excised, flushed gently with phosphate-buffered saline (PBS), and immersed in 10% formalin solution for histological analysis. The jejunum and ileum segments from three broilers in each group were opened longitudinally, washed with PBS and photographed using camera. The intestinal mucosa was scratched using a sterile glass slide. Cecal contents were collected for 16S rRNA sequencing. All the samples were preserved at −80°C until further measurements.

### Analysis of Intestinal Morphology

Intestine samples were fixed in 4% paraformaldehyde in PBS solution, dehydrated in ethanol, infiltrated with xylene and embedded in paraffin blocks. The paraffin sections were sliced and stained with Hematoxylin & Eosin (H&E). Photographs were obtained using the Nikon optical microscope (Tokyo, Japan). The villus length and crypt depth of each sample were randomly measured in 10 visual fields.

### Measurement of Levels of Serum Immune Parameters

The levels of immunoglobulins (IgA, IgM and IgY) and inflammatory factors (IL-1β, IL-4, IL-6, IL-10 and TNF-α) were analyzed following the manufacturer’s instructions (Huamei Biological Engineering Research Institute, Wuhan, China), and detected using a microplate reader (SynergyTM H1, BioTek, Winooski, VT, USA).

### Analysis of Serum Metabolomics

Metabolites were obtained from the serum samples (N = 6 in each group) using a methanol/water (4:1, v/v) solution. The mixture was settled at –20°C, crushed at 50 Hz for 6 min, and ultrasonicated at 40 kHz for 30 min at 5°C. Then, the samples were stood at –20°C for 30 min to precipitate the proteins. After centrifugation (13000 g, 15 min, 4°C), the supernatant was transferred to sample vials for LC-MS/MS detection.

2 μL of the separated samples were injected and detected on a Thermo UHPLC system equipped with an ACQUITY BEH C18 column (100 mm × 2.1 mm i.d., 1.7 µm; Waters Corporation, Milford, MA, USA). The mobile phases containing 0.1% formic acid in H_2_O and 0.1% formic acid in acetonitrile: isopropanol (1:1, v/v), and the flow rate was 0.4 mL/min. The mass spectrometric data were obtained using a Thermo UHPLC-Q Exactive Mass Spectrometer equipped with an electrospray ionization (ESI) source operating in positive or negative ion modes. Operating conditions: Aus gas heater temperature, 400°C; Aus gas flow rate, 30 psi; sheath gas flow rate, 40 psi; and ion-spray voltage floating, 3.5kV in positive mode and -2.8kV in negative mode. Detection was done over a mass range of 70-1050 m/z.

Raw data were preprocessed using Progenesis QI 2.3 (Nonlinear Dynamics, Waters Corporation, Milford, MA,USA). The metabolic features detected in > 80% of the samples were retained. After being filtered and normalized, the mass spectra of metabolic features were identified using the accurate mass, MS/MS fragment spectra and isotope ratio difference obtained by searching in reliable biochemical databases as Human Metabolome Database (HMDB) (http://www.hmdb.ca/) and Metlin database (https://metlin.scripps.edu/). Analysis for multivariate statistics was conducted using ropls (Version1.6.2, http://bioconductor.org/packages/release/bioc/html/ropls.html) package of R software from Bioconductor on a Majorbio cloud platform (https://cloud.majorbio.com). Principal component analysis (PCA) was performed by an unsupervised method. Partial least squares discriminant analysis (PLS-DA) was performed to detect and compare the metabolic changes between the groups, and the metabolite variables were scaled to pareto scaling. Variable importance in projection (VIP) scores were calculated using the PLS-DA model. Potential metabolic biomarkers were screened with a critical VIP > 1.0, *P* < 0.05, and fold change ≥ 1.2 or ≤ 0.833. Metabolites were clustered using hierarchical clustering, averaging, and Euclidean algorithms. The pathway classification was based on the Kyoto Encyclopedia of Genes and Genomes (KEGG) database (http://www.genome.jp/kegg/). Analysis for KEGG enrichment was performed on scipy.stats (Python packages) using Fisher’s exact test.

### Microbiological Analysis

The total DNA was extracted from the cecal samples using an E.Z.N.A.^®^ soil DNA Kit (Omega Bio-tek, Norcross, GA, USA), following the manufacturer’s instructions. The V3-V4 region of the 16S rRNA gene was selected to analyze the bacterial taxonomic composition, and PCR amplification was conducted using the custom barcoded universal primers 338F (5’-ACTCCTACGGGAGGCAGCA-3’) and 806R (5’-GGACTACHVGGGTWTCTA AT-3’). The PCR products were extracted and purified using an AxyPrep DNA Gel Extraction Kit (Axygen Biosciences, Union City, CA, USA). The purified amplicons were pooled in equimolar amounts and paired-end sequenced on the Illumina MiSeq platform (Illumina, San Diego, CA, USA), following the standard instructions of Majorbio Bio-Pharm Technology Co. Ltd. (Shanghai, China).

Raw data were quality-filtered by Trimmomatic and merged using FLASH1.2.1. Operational taxonomic units (OTUs) were selected by a threshold of 97% sequence similarity using UPARSE (version 7.1) with a novel ‘greedy’ algorithm that does chimera filtering and OTU clustering simultaneously. The bacterial taxonomy of 16S rRNA genes was conducted using an RDP Classifier algorithm (http://rdp.cme. msu.edu/) against the database with a threshold of 70%. α diversity was analyzed by Mothur1.30.2 (https://www.mothur.org/wiki/Download_ mothur). β diversity analysis was based on the unweighted UniFrac distance and was performed using QIIME1.9.1. The microbiota composition at different levels was determined based on tax_summary and R package version 3.3.1, and data were analyzed by one-way ANOVA and Tukey’s test. The Circos diagram was constructed using Circos-0.67-7 (http://circos.ca/). Analysis of the LDA effect size (LEfSe) was conducted to screen differentially abundant bacterial taxa at LDA score > 2.0. Microbial functions were predicted by Greengenes-based PICRUSt and differential analysis was performed by the STAMP software.

### Determination of VFA Production

The VFA concentrations were detected by the headspace sampler gas chromatography based on a previous study (22). Briefly, one gram of cecal samples was mixed with phosphorous acid, centrifuged at 12,000 g for 10 min at 4°C, and the supernatant was collected to measure the VFAs. The supernatant was injected into an Agilent Technologies GC7890 Network System with a flame ionization detector (Agilent Technologies, Wilmington, DE, USA).

### Statistical Analysis

Data are presented as mean ± SEM and were analyzed using SPSS (IBM SPSS 21.0, Chicago, IL, USA). The variations among groups were evaluated by One-way ANOVA and Tukey’s multiple comparison tests. Figures were generated using GraphPad Prism 8.0. Data for metabonomics and 16S rRNA sequencing were analyzed on the Majorbio Cloud Platform. Analyses for correlation assays were performed between serum metabolic biomarkers, differential microbials, weight loss, and levels of immune factors (immunoglobulins and inflammatory cytokines). Correlation coefficients were calculated using Pearson’s correlation distance, and heatmaps were conducted by R package to identify bivariate relationships between the variables. Significant differences were determined at *P* < 0.05.

## Results

### LA Alleviated the Weight Loss and Improved the Intestinal Barrier of LPS- Challenged Broilers

**Figure 1:**
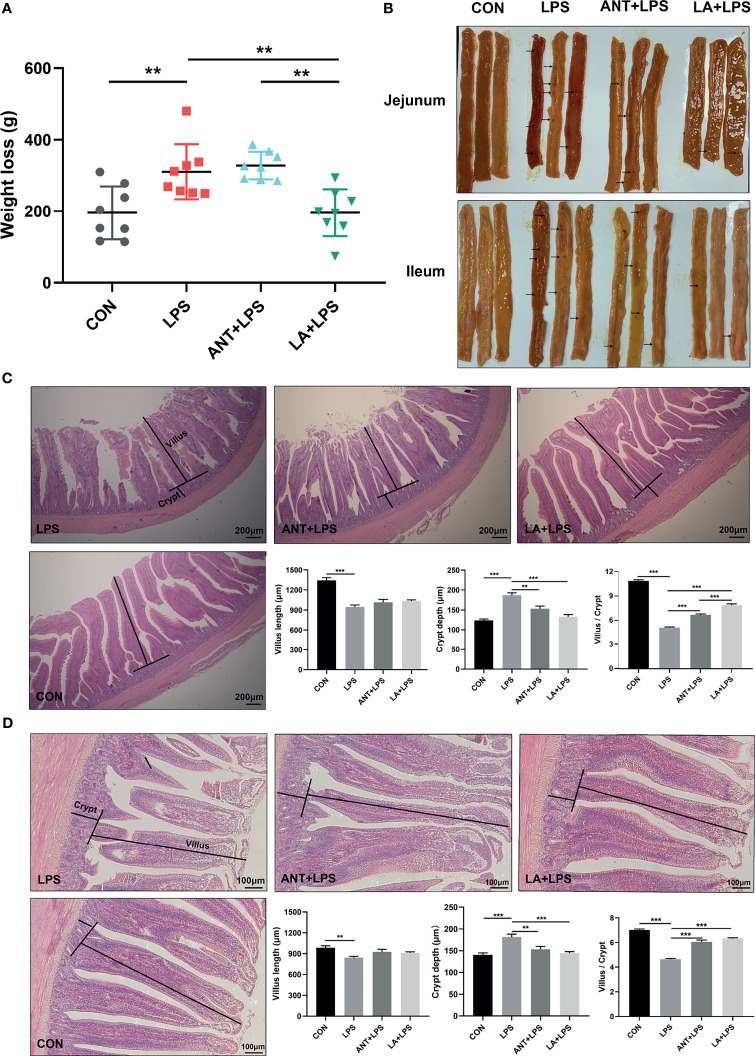
Lauric acid (LA) attenuated the weight loss and intestinal injuries of lipopolysaccharide (LPS)- challenged broilers. CON: fed with a basal diet and injected with saline; LPS: fed with a basal diet and injected with 0.5 mg/kg LPS; ANT+LPS: fed with a basal diet supplemented with 75 mg/kg ANT, and injected with 0.5 mg/kg LPS; LA+LPS: fed with a basal diet supplemented with 1000 mg/kg LA, and injected with 0.5 mg/kg LPS. N=8 in each group. **(A)** Weight loss. Weight loss was calculated using the equation: weight on day 42 - weight on day 44. **(B)** Pictures of the jejunal and ileal lumen. The arrows indicates the hemorrhagic spots. **(C)** Histomorphometric analysis of the jejunum by Hematoxylin & Eosin (H&E) staining. 40× magnification, scale bar: 200μm. **(D)** Ileum histomorphometric analysis. 100× magnification, scale bar: 100μm. The villus height and crypt depth shown in the pictures were randomly measured in 10 visual fields in each sample from each group. The data shown as mean ± SEM were analyzed using one-way ANOVA and Tukey’s test. **P < 0.01, ***P < 0.001.

As shown in **Figure 1A**, LPS challenge dramatically increased the weight loss in broilers in comparison to that in the untreated group (*P* < 0.01). LA supplementation significantly inhibited the LPS-induced weight loss and reversed it to the basal level (*P* < 0.01), whereas ANT pretreatment had no effect (*P* > 0.05). The morphology of jejunum and ileum was displayed in **Figure 1B**. Obvious hemorrhagic spots were found in both intestinal parts after LPS challenge, while LA and ANT pretreatments alleviated the injuries. H&E staining revealed that the intestinal epithelial villi were severely damaged in response to LPS (**Figures 1C, D**), as evidenced by the broken villi structure and mucosal layer erosion. LA administration decreased the degree of tissue injury, and significantly reduced the crypt depth, and increased the villus/crypt ratio both in jejunum and ileum (*P* < 0.001) (**Figures 1C, D**). These results suggest that LA supplementation exerts a protective role in LPS- challenged broilers by reducing the weight loss and enhancing mucosal structures.

### LA Promoted the Immune Responses and Inhibited the Inflammation in LPS- Challenged Broilers

**Figure 2:**
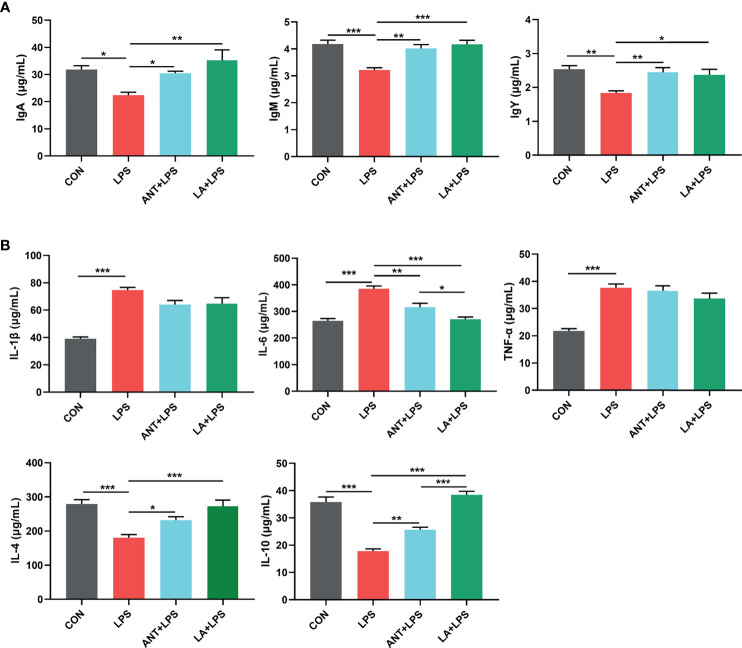
Lauric acid (LA) modulated the immune functions of broilers under lipopolysaccharide (LPS)- challenge. CON: fed with a basal diet and injected with saline; LPS: fed with a basal diet and injected with 0.5 mg/kg LPS; ANT+LPS: fed with a basal diet supplemented with 75 mg/kg ANT, and injected with 0.5 mg/kg LPS; LA+LPS: fed with a basal diet supplemented with 1000 mg/kg LA, and injected with 0.5 mg/kg LPS. **(A)** Concentrations of serum IgA, IgM, and IgY **(B)** Levels of serum cytokines (IL-1β, IL-4, IL-6, IL-10, and TNF-α). Data shown as mean ± SEM were analyzed by one-way ANOVA and Tukey’s test (N = 8 in each group). *P < 0.05, **P < 0.01, ***P < 0.001.

As displayed in **Figure 2A**, LPS treatment dramatically decreased the concentrations of serum IgA, IgM and IgY (*P* < 0.05, *P* < 0.001 and *P* < 0.01, respectively), whereas LA and ANT significantly reversed this reducing trend. **Figure 2B** shows the expressions of inflammatory cytokines. Broilers exposed to LPS exhibited a marked increase of the proinflammatory cytokines (IL-1β, IL-6 and TNF-α) (*P* < 0.001). LA and ANT pretreatments remarkably reduced the upregulated levels of IL-6 (*P* < 0.001 and *P* < 0.01, respectively), but showed no significant effects on IL-1β and TNF-α levels (*P* > 0.05). The concentrations of anti-inflammatory cytokines (IL-4 and IL-10) were dramatically reduced under LPS challenge (*P* < 0.001), while LA supplementation markedly reversed this trend (*P* < 0.001). These results indicate that LA has a strong capacity to enhance immunity and inhibit inflammation.

### Serum Metabolic Profiling Analysis by Untargeted HPLC/MS Metabolomics

**Figure 3:**
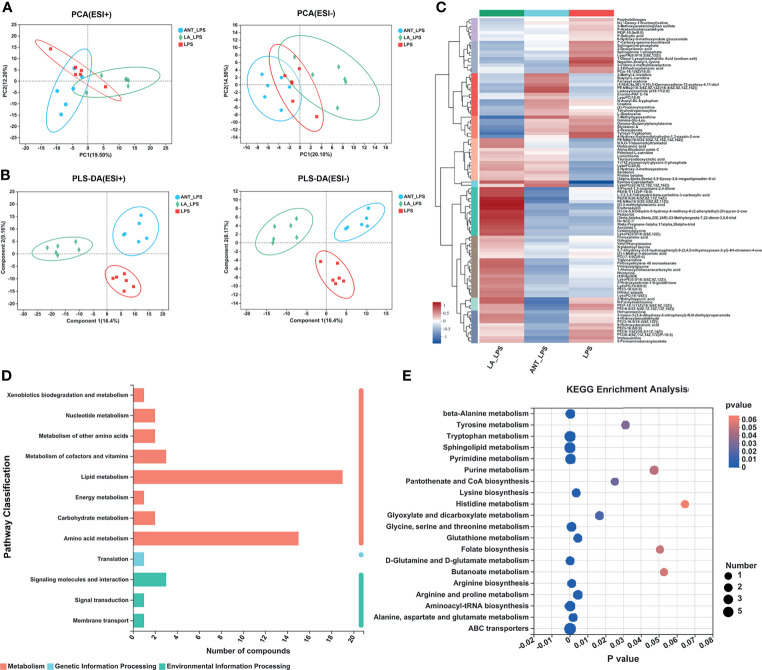
Multivariate statistical analysis, hierarchical clustering and summary of Kyoto Encyclopedia of Genes and Genomes (KEGG) enrichment pathways. LPS: fed with a basal diet and injected with 0.5 mg/kg LPS; ANT+LPS: fed with a basal diet supplemented with 75 mg/kg ANT, and injected with 0.5 mg/kg LPS; LA+LPS: fed with a basal diet supplemented with 1000 mg/kg LA, and injected with 0.5 mg/kg LPS. N=6 in each group. **(A)** Principal component analysis (PCA) score plot of nontargeted metabolite profiling of the serum samples among the three groups in both positive (ESI+) and negative ionization modes (ESI-). **(B)** Partial least squares-discriminant analysis (PLS-DA) score plot of metabolite profiling among groups in ESI+ and ESI-. **(C)** Hierarchical clustering analysis of serum metabolites from the LPS, ANT+LPS and LA+LPS groups. **(D)** KEGG pathway classification. The compounds were enriched and numbered at the second KEGG level. **(E)** Bubble diagram showing the KEGG enrichment analysis. The bubble size indicates enriched the numbers, while the color shade indicates the differences.

Multivariate analyses, including PCA and PLS-DA, were performed to uncover the clustering trends in each group. The results in the PCA score plot showed obvious changes in serum metabolites among the three groups in both ESI+ (PC1 = 19.50%, PC2 = 12.20%) and ESI- (PC1 = 20.10%, PC2 = 14.40%) (**Figure 3A**). The supervised pattern recognition of PLS-DA displayed class-discriminating variations. As shown in **Figure 3B**, significant variations in ESI+ were obtained in the serum metabolomes (component1 = 16.4%, component2 = 9.15%), while ESI- also showed complete separation (component1 = 16.4%, component2 = 8.17%). The ariation tendencies of serum metabolites are displayed in a hierarchical clustering heatmap in **Figure 3C**, suggesting significant variations of LA+LPS from LPS and ANT+LPS groups.

### Identification of Serum Metabolic Biomarkers

As summarized in **Table 2**, the identified 27 biomarkers belong to the categories lipids and lipid-like molecules (11 metabolites), benzenoids (3), phenylpropanoids and polyketides (2), organoheterocyclic compounds (4), organic acids and derivatives (1), and others (6). There were 13 significantly different compounds in the LA+LPS vs. LPS group. Twelve metabolites were significantly increased, including 3,8-dihydroxy-6-methoxy-7(11)-eremophilen-12,8-olide, PS(14:1(9Z)/18:0), Mactraxanthin, 12-Hydroxy-8,10-octadecadienoic acid, 3,4-Dimethyl-5-pentyl-2-furanpentadecanoic acid, Monoisobutyl phthalic acid, 1-H-Inden-1-one,2,3-dihydro-3,3,5,6-tetramethyl, Daidzein, Indole-3-carbinol, Spirolide B, PGPC and PAz-PC, while only one was down-regulated, namely, 2’’,4’’,6’’-Triacetylglycitin. Twenty-two biomarkers were significant in the LA+LPS vs. ANT+LPS comparison, and these are mainly belonged to the lipids and lipid-like molecules (three increased and seven decreased metabolites). Other significant metabolites were in benzenoids, phenylpropanoids and polyketides, organoheterocyclic compounds, and the unclassified categories. The above findings suggest that LA pretreatment has a strong capacity to modulate the serum metabolome, particularly the lipid metabolism.

**Table 2:** Comparisons of Serum metabolic biomarkers.

Number	Metabolites	Formula	Category	Mass(M/Z)*^2^*	RT(Min)*^1^*	LA+LPS VS LPS		LA+LPS VS ANT+LPS	
						FC	*P* value	FC	*P* value
1	3,8-Dihydroxy-6-methoxy-7(11)-eremophilen-12,8-olide	C16H24O5	Lipids and lipid-like molecules	331.1299	3.5549	1.2581*	0.0436	1.2432	0.0663
2	PS(14:1(9Z)/18:0)	C38H72NO10P	Lipids and lipid-like molecules	756.4795	9.2650	1.3076**	0.0021	1.3434***	0.0000
3	Mactraxanthin	C40H60O6	Lipids and lipid-like molecules	678.4710	9.1518	1.2785**	0.0023	1.1194**	0.0023
4	12-Hydroxy-8,10-octadecadienoic acid	C18H32O3	Lipids and lipid-like molecules	295.2275	8.3016	1.2530*	0.0224	1.4020*	0.0164
5	3,4-Dimethyl-5-pentyl-2-furanpentadecanoic acid	C26H46O3	Lipids and lipid-like molecules	451.3427	9.1118	1.2052*	0.0427	1.3609*	0.0147
6	Lactosylceramide (d18:1/12:0)	C42H79NO13	Lipids and lipid-like molecules	788.5444	9.3136	0.9707	0.7236	0.7928*	0.0327
7	3-hydroxytetradecanoyl carnitine	C21H41NO5	Lipids and lipid-like molecules	370.2956	6.3694	0.9406	0.3435	0.7980*	0.0152
8	Quinquenoside F1	C42H74O15	Lipids and lipid-like molecules	836.5421	9.3136	1.0397	0.5084	0.8283*	0.0446
9	PS(18:3(9Z,12Z,15Z)/20:0)	C44H80NO10P	Lipids and lipid-like molecules	814.5597	9.4268	0.9547	0.5517	0.7819*	0.0301
10	PS(14:0/24:1(15Z))	C44H84NO10P	Lipids and lipid-like molecules	840.5743	9.6857	0.9879	0.7992	0.8057*	0.0263
11	PE-NMe2(16:0/18:4(6Z,9Z,12Z,15Z))	C41H74NO8P	Lipids and lipid-like molecules	772.5495	9.6372	0.9924	0.9230	0.8021*	0.0390
12	Monoisobutyl phthalic acid	C12H14O4	Benzenoids	205.0861	7.0005	1.5611***	0.0000	1.0311	0.0744
13	1-H-Inden-1-one,2,3-dihydro-3,3,5,6-tetramethyl	C13H16O	Benzenoids	233.0922	1.6437	1.2735*	0.0229	1.4919***	0.0000
14	2-cyano-3-(3,4-dihydroxy-5-nitrophenyl)-N,N-diethylpropanamide	C14H17N3O5	Benzenoids	325.1509	1.0932	1.0824	0.3580	1.2394*	0.0339
15	2’’,4’’,6’’-Triacetylglycitin	C28H28O13	Phenylpropanoids and polyketides	614.1822	5.6900	0.7859**	0.0065	0.7612**	0.0028
16	Daidzein	C15H10O4	Phenylpropanoids and polyketides	255.0654	3.4566	1.2876*	0.0256	1.3217*	0.0146
17	Indole-3-carbinol	C9H9NO	Organoheterocyclic compounds	189.1021	1.5305	1.2283*	0.0328	0.9617	0.7315
18	Plantagonine	C10H11NO2	Organoheterocyclic compounds	178.0863	2.3075	1.1020	0.1699	1.2178**	0.0099
19	Spirolide B	C42H63NO7	Organoheterocyclic compounds	694.4662	8.9901	1.3447**	0.0034	1.1670	0.0009
20	Hydroxynalidixic acid	C12H12N2O4	Organoheterocyclic compounds	231.0765	1.1904	1.1852	0.1249	1.2832*	0.0313
21	Hydroxyphenylacetylglycine	C10H11NO4	Organic acids and derivatives	208.0608	1.5246	1.0658	0.1244	1.2213**	0.0022
22	Alanylclavam	C8H12N2O4	Others	201.0871	0.8988	1.1186	0.1095	1.2082*	0.0128
23	Nicotyrine	C10H10N2	Others	159.0917	2.3075	1.1694	0.1763	1.3194*	0.0288
24	2-Ethylacrylylcarnitine	C12H21NO4	Others	244.1545	3.2139	0.9204	0.3214	0.7578*	0.0175
25	(3-Arylcarbonyl)-alanine	C10H11NO3	Others	194.0813	1.4333	1.1501	0.0756	1.3030**	0.0031
26	PGPC	C29H56NO10P	Others	608.3567	8.1695	1.3652**	0.0066	1.2813***	0.0006
27	PAz-PC	C33H64NO10P	Others	666.4351	8.4563	1.2456**	0.0068	1.1313	0.0026

^1^RT means retention time. ^2^FC is the fold change (differential multiple) of the metabolite expression between groups (LA vs. LA+LPS, LA+LPS vs. ANT+LPS). *P < 0.05, **P < 0.01, ***P < 0.001.

### Analysis of Differential Metabolic Pathways

KEGG pathway classification revealed that most serum compounds were involved in metabolism, particularly in lipid metabolism and amino acid metabolism (**Figure 3D**). The bubble diagram indicates the number of metabolites enriched in the KEGG signaling pathways (**Figure 3E**). Results showed that there were 17 significantly abundant pathways including Sphingolipid metabolism (number of metabolites = 7, *P* = 0), ABC transporters (7, *P* = 0.0001), Tryptophan metabolism (6, *P* = 0.0001), Pyrimidine metabolism (5, *P* = 0.0003), Aminoacyl-tRNA biosynthesis (5, *P* = 0.0001), Arginine and proline metabolism (4, *P* = 0.0044), Glycine, serine and threonine metabolism (4, *P* = 0.0009), beta-alanine metabolism (4, *P* = 0.0002), Purine metabolism (3, *P* = 0.0472), Tyrosine metabolism (3, *P* = 0.0311), Glyoxylate and dicarboxylate metabolism (3, *P* = 0.0164), Glutathione metabolism (3, *P* = 0.0002), Lysine biosynthesis (3, *P* = 0.0035), Alanine, aspartate and glutamate metabolism (3, *P* = 0.0018), Arginine biosynthesis (3, *P* = 0.001), D-Glutamine and D-glutamate metabolism (3, *P* = 0.0001), Pantothenate and CoA biosynthesis (2, *P* = 0.025).

**Figure 4:**
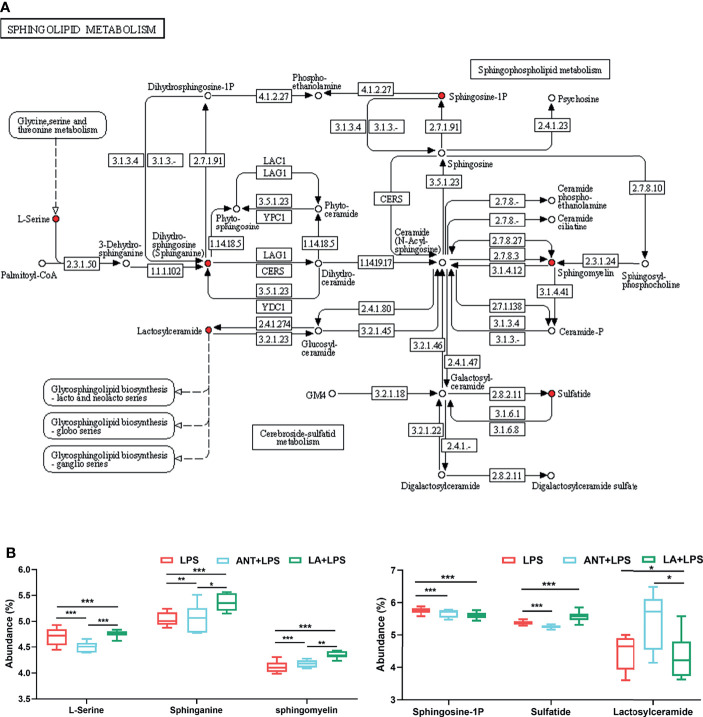
Analysis of Sphingolipid metabolism pathway. LPS: fed with a basal diet and injected with 0.5 mg/kg LPS; ANT+LPS: fed with a basal diet supplemented with 75 mg/kg ANT, and injected with 0.5 mg/kg LPS; LA+LPS: fed with a basal diet supplemented with 1000 mg/kg LA, and injected with 0.5 mg/kg LPS. N=6 in each group. **(A)** Kyoto Encyclopedia of Genes and Genomes (KEGG) pathway map of Sphingolipid metabolism (map00600). The red nodes represent the differential metabolites. **(B)** Analysis of the differential metabolites by one-way ANOVA and Tukey’s test. *P < 0.05, **P < 0.01, ***P < 0.001.

The dominant pathway was Sphingolipid metabolism (*P* < 0.001), and LA supplementation significantly modulated the expression of six metabolites involved in this pathway (map00600) (**Figure 4A**). Specifically, as shown in **Figure 4B**, compared to LPS group, LA markedly increased the abundance of L-Serine, Sphinganine, Sphigomyelin and Sulfatide (*P* < 0.001), and decreased the abundance of Sphingosine-1P and Lactosylceramide (*P* < 0.001 and *P* < 0.05, respectively).

### Analysis of Cecal Microbial Composition

**Figure 5:**
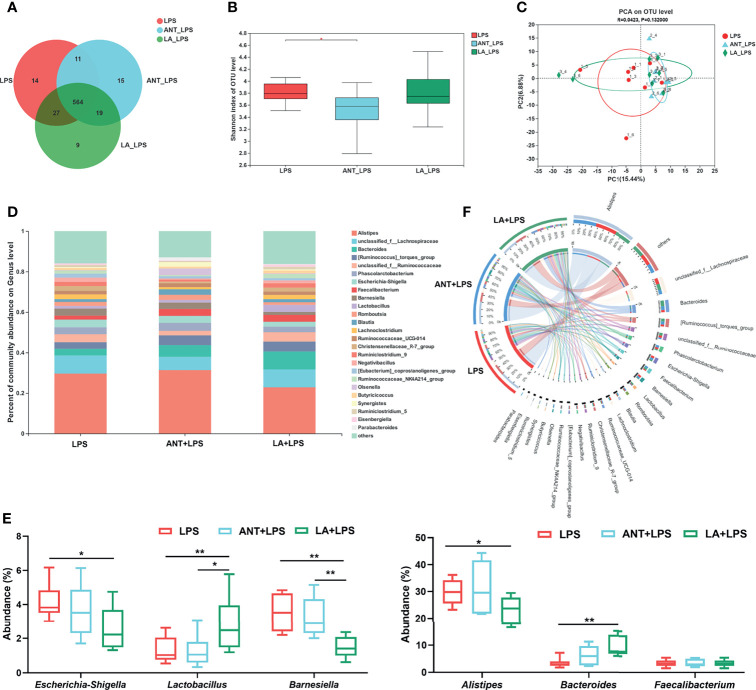
Analysis of the diversity and composition of gut microbiota. LPS: fed with a basal diet and injected with 0.5 mg/kg LPS; ANT+LPS: fed with a basal diet supplemented with 75 mg/kg ANT, and injected with 0.5 mg/kg LPS; LA+LPS: fed with a basal diet supplemented with 1000 mg/kg LA, and injected with 0.5 mg/kg LPS. N=8 in each group. **(A)** Venn diagram presenting the operational taxonomic units (OTUs) from each group. **(B)** Shannon index of OTU level reflecting the α diversity. **(C)** β diversity shown in a Principal component analysis (PCA) scatterplot. **(D)** Bar graph of microbial composition at the genus level. **(E)** Box plot of the significant genera among groups. **(F)** Circos diagram indicating the dominant genera in each group. The data were analyzed by one-way ANOVA and Tukey’s test. *P < 0.05, **P < 0.01.

We investigated the alterations in the cecal microbiota of LPS-challenged broilers. The total OTUs in the LPS, ANT+LPS and LA+LPS groups were 616, 609 and 619, respectively, and LA pretreatment exhibited fewer unique OTUs (9) than the other two groups (**Figure 5A**). ANT pretreatment significantly decreased Shannon index that represents α diversity (*P* < 0.05), while no obvious changes were found in LA- pretreated broilers (*P* > 0.05) (**Figure 5B**). β diversity displayed in a PCA scatterplot indicated an obvious shift of the LA+LPS and ANT+LPS groups from the LPS group (**Figure 5C**). **Figures 5D, E** show the differences in microbial compositions at the genus level. LA pretreatment significantly reduced the abundance of *Escherichia-Shigella* but dramatically increased the richness of *Lactobacillus* when compared to that of the LPS-challenged group (*P* < 0.05 and *P* < 0.01, respectively). LA pretreatment also induced a marked downregulation of *Barnesiella* and *Alistipes* (*P* < 0.01 and *P* < 0.05, respectively). The abundance of *Bacteroides* was markedly increased in the LA+LPS group compared to that of the LPS group (*P* < 0.01), whereas no significant changes of *Faecalibacterium* were observed among the three groups (*P* > 0.05). The Circos diagram shows a significantly higher proportion of *Bacteroides* and *Lactobacillus* in the LA+LPS group than the other two groups. *Olsenella* was the dominant genus in the ANT+LPS group, while *Escherichia-Shigella* and *Barnesiella* were dominant in the LPS group (**Figure 5F**).

### Taxonomic Biomarkers, Predicted Functions, and VFA Production of Cecal Microbiota

**Figure 6:**
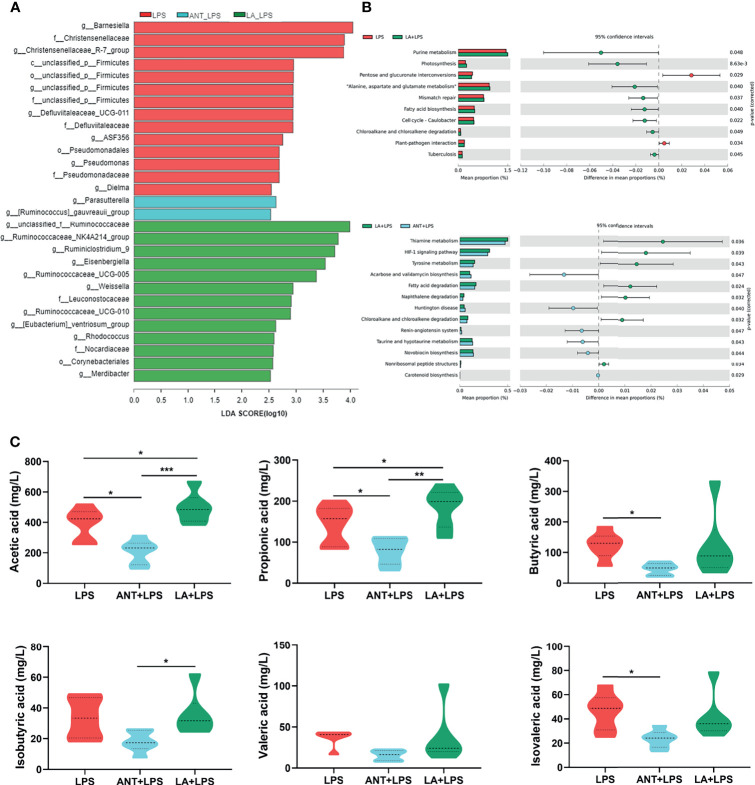
Analysis of taxonomic biomarkers, predicted microbial functions and volatile fatty acid (VFA) production. LPS: fed with a basal diet and injected with 0.5 mg/kg LPS; ANT+LPS: fed with a basal diet supplemented with 75 mg/kg ANT, and injected with 0.5 mg/kg LPS; LA+LPS: fed with a basal diet supplemented with 1000 mg/kg LA, and injected with 0.5 mg/kg LPS. **(A)** Histogram of Linear discriminant analysis (LDA) scores representing the taxonomic biomarkers by LDA effect size (LEfSe) analysis. LDA score (log10) >2 suggests the enriched taxa in cases, N=8 in each group. **(B)** Comparisons of gut microbes (LPS vs. LA+LPS, LA+LPS *vs*. ANT+LPS) by statistical analysis of taxonomic and functional profiles (STAMP), N=8 in each group. **(C)** Violin plots displaying VFA production of the cecal microbiota, N=6 in each group. Data shown as Mean ± SEM were analyzed by one-way ANOVA and Tukey’s test. *P < 0.05, **P < 0.01, ***P < 0.001.

As shown in **Figure 6A**, the taxonomic biomarkers were the genera *Barnesiella*, *Christensenellaceae*_R-7_group, unclassified_p_Firmicutes, *Defluviltaleaceae*_UCG-011, ASF356, *Pseudomonadaceae*, *Dielma* and *Parasutterella* in the LPS group. ANT pretreatment markedly increased the abundance of g_*Parasutterella*, and g_[*Ruminococcus*]_ *gauvreauii*_group. In the LA+LPS group, the predominant bacteria belonged to *Ruminococcaceae* including the genera unclassified_f_*Ruminococcaceae*, *Ruminococcacea*_NK4A214_group, *Ruminiclostridium*_9, *Ruminococcacea*_ UCG-005 and *Ruminococcacea*_UCG-010). Additionally, g_*Eisenbergiella*, g_*Weissella*, f_*Leuconostocaceae*, f_*Nocardiaceae*, O_*Corynebacteriales* and g_*Merdibacter* were also enriched in the LA+LPS group. Results in **Figure S1A** revealed high-level phylogenetic alterations in the taxonomic biomarkers between the LPS and LA+LPS groups.

The heatmap of Greengenes-based PICRUSt analysis showed that the most abundant functional genes were in metabolic pathways, biosynthesis of secondary metabolites, biosynthesis of amino acids and carbon metabolism, microbial metabolism in diverse environments (**Figure S1B**). STAMP analysis revealed that LA pretreatment induced higher richness of functional genes for purine metabolism, photosynthesis, “Alanine, aspartate and glutamate metabolism”, mismatch repair, fatty acid biosynthesis, cell cycle-caulobacter, chloroalkane and chloroalkene degradation and tuberculosis than those of the LPS group. LA also significantly increased the abundance of genes related to biosynthesis and decreased the abundance of genes involved in amino acid and lipid metabolism, compared to those in ANT pretreatment (**Figure 6B**).

**Figure 6C** shows the levels of cecal VFA production. LA supplementation markedly increased the concentrations of acetic and propionic acids in comparison to those in the LPS-treated group (*P* < 0.05), whereas no significant differences in butyrate acid, isobutyric acid, valerate and isovalerate (*P* > 0.05). ANT dramatically decreased the concentration of VFAs, including acetic acid, propionic acid and isovalerate (*P* < 0.05).

### Pearson’s Correlation Analysis

**Figure 7:**
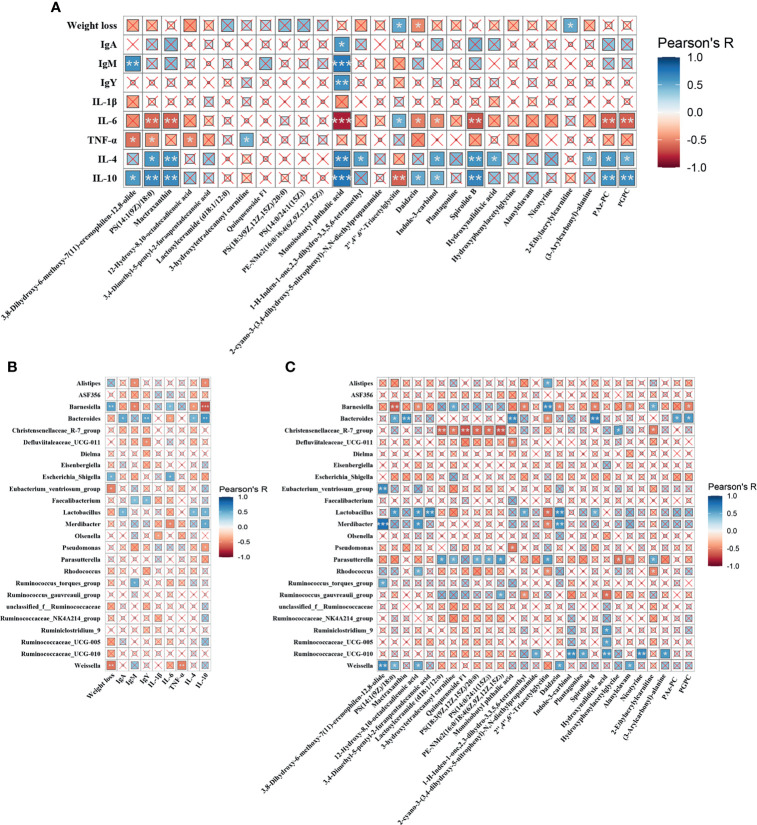
Heatmaps of Pearson’s correlation. **(A)** Correlation between the metabolic biomarkers and immune indices. **(B)** Correlation between differential microbiota and immune parameters. **(C)** Correlation between the differential microbiota and metabolic biomarkers. The sizes of the small boxes reflect the correlation coefficient. The colors were according to the Pearson’s correlation coefficient distribution. ×P > 0.05, *P < 0.05, **P < 0.01, ***P < 0.001.

The correlations among metabolites, gut microbes and immune functions were examined to further confirm the underlying mechanisms. As shown in **Figure 7A**, Monoisobutyl phthalic acid showed the strongest positive correlation with IgA, IgM, IgY, IL-4, and IL-10, and negatively linked with IL-6. Similar trends (correlated to IL-4, IL-10 and IL-6) were also found in metabolic biomarkers, including PS(14:1(9Z)/18:0), Mactraxanthin, Indole-3-carbinol, Spirolide B, PAz-PC and PGPC, while 2’’,4’’,6’’-Triacetylglycitin. Weight loss showed a positive relationship with 2’’,4’’,6’’-Triacetylglycitin and 2-Ethylacrylylcarnitine, and a negative relationship with Daidzein. **Figure 7B** displays the correlation between the differential cecal microbes and immune indices. The results showed that the genera *Alistipes*, *Barnesiella*, *Escherichia_Shigella* and *Weissella* were negatively linked with immunoglobulins and anti-inflammatory cytokines and, were strongly correlated with proinflammatory cytokines. Conversely, *Bacteroides*, *Faecalibacterium, Lactobacillus* and *Merdibacter* showed the opposite trend. **Figure 7C** shows that *Barnesiella*, and *Christensenellaceae*_R-7_group were mainly negatively correlated with the metabolic biomarkers, while *Bacteroides*, *Lactobacillus*, *Parasutterella*, *Ruminococcaceae*_UCG-010 and *Weissella* were positively correlated with the metabolites.

## Discussion

Bacterial LPS challenge is a serious problem in animal production which ultimately affects the quality and safety of animal-derived foods (23). Previous studies have shown that LPS challenge led to a compromised growth performance of broilers (24). Similarly, we found that LPS exposure resulted in a significant weight loss, whereas LA reversed this change. The intestine is believed to be the main target organ for LPS. It can destroy the intestinal mucosal barrier, increase gut permeability and induce intestinal inflammation (25, 26). In this study, LPS treatment significantly caused intestinal mucosal injuries, whereas LA attenuated these detrimental effects, suggesting the capacity of LA to support the integrity of intestinal structures. The regulation and repair of the intestinal barrier by MCFAs have been proven by many studies as they can modulate the expression of tight junction proteins (27, 28). Consistently, LA supplementation prevented the intestinal barrier dysfunction of mice exposed to enterohemorrhagic *Escherichia coli* infection (29).

Improved immune functions are essential for animals to render the body free from pathogens. LA and its monoglycerides have been demonstrated to possess immuno-modulatory properties. They can modulate the functions of immune cells by interacting cell membrane and activating signaling pathways (30). IgA, IgM and IgY are the main animal immunoglobulins against infection. This study revealed that LA markedly inhibited the decreased levels of LPS-induced decrease in IgA, IgM and IgY levels, indicating the immune promotion effects of LA supplementation. Consistently, it was reported that supplementation with MCFAs or organic acid significantly improved IgG, IgA and IgM concentrations in piglets (31, 32). LPS exposure can activate excessive immune responses and induce immune stress. This process is mainly orchestrated by the overproduction of pro-inflammatory cytokines, particularly IL-1, IL-6 and TNF-α (33). We found that LPS challenge led to a significant increase of these cytokines, whereas LA reversed this trend. Moreover, LA treatment significantly increased the levels of anti-inflammatory cytokines (IL-4 and IL-10), which play a critical role in limiting inflammation in immune-mediated pathologies. Therefore, our results indicated the ability of LA to suppress LPS-induced inflammation. Similarly, the anti-inflammatory effects of LA were confirmed by many previous studies, where LA supplementation decreased IL-6 and TNF-α in mice after *Escherichia coli* challenge (29); orally administered glyceryl monolaurate could be useful in reducing gut inflammation (34).

We demonstrated that LA can modulate metabolism, particularly lipid metabolism. Dietary LA is normally transported directly to the liver and metabolized for energy in the mitochondria. It plays an essential role in regulating fatty acid homeostasis. It has been reported that LA has a strong capacity in the prevention and treatment of obesity by altering serum lipid metabolites such as sphingomyelin and lysophosphatidylcholine (18). Yang et al. found that LA increased the levels of lipid metabolites, including sphingomyelin and triglycerides in lactating mice (35). In our study, LA significantly upregulated the abundance of lipids compared to that of the LPS and ANT+LPS group. The correlation analysis revealed that PS (14:1(9Z)/18:0) and Mactraxanthin were positively correlated with IL-4 and IL-10, and were negatively correlated with IL-6. PS has been reported to exert an anti-inflammatory activity by downregulating proinflammatory molecules (36, 37), whereas Mactraxanthin is a carotenoid with antioxidant, hepatoprotective and membrane stabilizing properties (38). Hence, our results indicate that the upregulation of these two lipids might have contributed in alleviating LPS-induced inflammation. Furthermore, LA could significantly modulate Sphingolipid metabolism by increasing L-Serine, Sphinganine, Sphingomyelin, and Sulfatide, and decreasing Sphingosine-1P and Lactosylceramide. L-Serine and Sphinganine are the initial substrates and the other four are the final metabolites in Sphingolipid metabolism. Sphingolipid metabolites play a crucial role in inflammatory signaling. Several studies have demonstrated that Sphingomyelin and Sulfatide possess strong capacity to suppress inflmmation. For example, dietary Sphingomyelin could protect against dysfunctional lipid metabolism, gut dysbiosis, and inflammation both *in vivo* and *in vitro* (39); and Sulfatide decreased the proinflammatory cytokines (IL-6, IL-8 and TNF-α) in human adipose tissue (40). Sphingosine-1P is crucial in regulating cell growth and survival, but it is also recognized as a critical activator of pro-inflammatory and inflammation-associated cancer progression (41, 42). Similarly, Lactosylceramide can induce inflammation and is regarded as a potential biomarker of many diseases, such as inflammatory bowel disease and neuroinflammatory disease (43–45). Therefore, the increased Sphingomyelin and Sulfatide, and the decreased Sphingosine-1P and Lactosylceramide revealed the anti-inflammatory effects of LA. These results suggested that LA might suppress LPS-induced inflammation by modulating the lipid and Sphingolipid metabolism.

The gut microbiota is a biological barrier against the colonization of pathogenic bacteria, and its alterations play a pivotal role in the pathogenesis of diseases (46). LPS challenge can disrupt the ecological balance in the gut microbiota and cause gut dysbiosis (47). In this study, the abundance and composition of the cecal microbiota were improved by LA supplementation. LA pretreatment reduced the abundance of *Escherichia-Shigella*, *Barnesiella* and *Alistipes*, and increased that of *Lactobacillus* and *Bacteroides*, which was further confirmed by Circos analysis. *Escherichia-Shigella* belongs to the phylum Proteobacteria and can become pathogenic bacteria when stimulated by stress, leading to an increased intestinal permeability, disrupted epithelial barriers and intestinal diseases (48, 49). Our results also showed positive correlations between *Escherichia-Shigella*, weight loss and IL-6. The results are in line with those by Rubio et al. who concluded that *Escherichia-Shigella* negatively correlated with weight gain and final weight of broilers (50); *Escherichia-Shigella* were positively correlated with IL-6 in rats with type 2 diabetes (51). Therefore, the decreased abundance of *Escherichia-Shigella* following LA pretreatment indicated the protective effects of LA against LPS exposure. *Barnesiella*, a genus of the family Porphyromonadaceae, within the phylum Bacteroidetes, is one of the most abundant genera in the intestine. Studies have demonstrated its functions in the elimination of intestinal pathogens and modulation of immune responses (52, 53). By contrast, our results revealed a negative effect of *Barnesiella* as it showed a highly positive link with weight loss and IL-6, and a remarkably negative correlation with IgM, IL-4, IL-10 and anti-inflammatory metabolite PS(14:1(9Z)/18:0). Consistently, it was reported that *Barnesiella* was significantly increased in fasting hyperglycemia and in high plasma concentrations of proinflammatory cytokines in mice (54). *Alistipes* is a new genus belonging to the Bacteroidetes phylum. It is a potential opportunistic pathogen in diseases, such as liver fibrosis, colorectal cancer, cardiovascular disease and mood disorders (55). Studies have showed that it is highly relevant to dysbiosis and inflammation (56). Thus, the decreased abundance of *Alistipes* in LA pretreatment suggested an improved microbiota. *Lactobacillus* is a well-known probiotic in the gut. It provides various benefits to the host including inhibiting pathogens and exerting anti-inflammatory effects (57, 58). Accordingly, it has been reported by a previous study that supplemented with coconut oil significantly increased the abundance of *Lactobacillus reuteri* in mice (59). Moreover, LA upregulated the richness of *Bacteroides*, while this bacteria showed a positive correlation with IgA, IgY, IL-4, IL-10, PS(14:1(9Z)/18:0) and Mactraxanthin, and negatively with IL-6. *Bacteroides* strains are gram-negative bacteria belonging to the phylum Bacteroidetes. Many studies have revealed their functions in digesting various types of polysaccharides and in maintaining the immune system (60, 61). *Bacteroides* strains have been reported to exhibit a marked anti-inflammatory activity against *E.coli* LPS-induced IL-8 release (62), suggesting that an increased abundance of *Bacteroides* might contribute in suppressing inflammatory responses.

VFAs are important metabolites of the gut microbiota to maintain the intestinal homeostasis, as they can provide energy for intestinal epithelial cells, activate anti-inflammatory signaling cascades and strengthen gut barrier functions (63). In this study, LA markedly increased the concentrations of acetic and propionic acids upon LPS exposure. These two acids are the predominant VFAs that were produced by gut microbiota. Numerous studies have demonstrated the anti-inflammatory and immunomodulatory functions of them both *in vivo* and *in vitro.* For instance, acetic and propionic acids modulated the production of inflammatory mediators and enhanced phagocytosis of immune cells against infectious bacteria (64); mixtures of acetate, propionate and butyrate suppressed the TNF-α- induced proinflammatory signaling in Caco-2 cells and mouse colons (65); indole propionic acid, which were produced by gut microbiota, showed strong anti-inflammatory and antioxidant properties (66). Thus, the increased concentrations of VFAs induced by LA supplementation might contribute to alleviating LPS- induced inflammation. Moreover, the LefSe analysis revealed that the taxonomic biomarkers in LA+LPS group mainly belonged to the family Ruminococcaceae. It was reported that Ruminococcaceae is the main VFA producing-bacteria in the gut which can degrade polysaccharides (67); previous studies found that Ruminoccaceae can directly inhibit *Clostridium difficile* infection (68). The dominance of Ruminococcaceae further indicated the protective role of LA during LPS challenge.

## Conclusions

In summary, this study showed that LA protects against LPS-induced inflammation and injuries in broilers, and possibly mediated by enhancing the gut barrier and immune responses, modulating lipid metabolism, and altering gut microbiota and VFA production. The findings suggest the potential of LA to reduce antibiotic usage in animal production and improve food safety.

## Data Availability Statement

The raw data supporting the conclusions of this article will be made available by the authors, without undue reservation, to any qualified researcher. The 16S rRNA sequencing data can be found here: http://www.ncbi.nlm.nih.gov/bioproject/754808; PRJNA754808.

## Ethics Statement

The animal study was reviewed and approved by the Animal Care and Use Committee of Zhejiang Agricultural and Forestry University.

## Author Contributions

CY designed and supervised the study. YPW drafted the manuscript. QL, YX, RZ, and YY conducted the experiments. YPW, JL, and YL performed data analysis. CY and YXW revised the manuscript. All authors have read and approved the final version of the manuscript.

## Funding

The present study was supported by Zhejiang Provincial Leading Innovation and Entrepreneurship Team Project (No. 2020R01015), Zhejiang Key Agricultural Research Institute of Green Animal Health Products Project (No. 2021Y30004), Zhejiang Provincial Key Research and Development Program (No. 2019C02051 and No. 2020C02032), and the National Natural Science Foundation of China (No. 32002212).

## Conflict of Interest

Authors JL and YL are employed by Zhejiang Vegamax Biotechnology Co., Ltd.

The remaining authors declare that the research was conducted in the absence of any commercial or financial relationships that could be construed as a potential conflict of interest.

## Publisher’s Note

All claims expressed in this article are solely those of the authors and do not necessarily represent those of their affiliated organizations, or those of the publisher, the editors and the reviewers. Any product that may be evaluated in this article, or claim that may be made by its manufacturer, is not guaranteed or endorsed by the publisher.

## Supplementary Material

Supplementary Figure 1
**(A)** Phylogenetic tree of gut microbiota**. (B)** Heatmap of the predicted functions by PICRUSt analysis.
