# Supplementary figures and images for: Integrating Serum Metabolome and Gut Microbiome to Evaluate the Benefits of Lauric Acid on Lipopolysaccharide- Challenged Broilers
Source: Front Immunol. 2021 Oct 15;12:759323. doi: 10.3389/fimmu.2021.759323 (PMC8554146; doi:10.3389/fimmu.2021.759323)

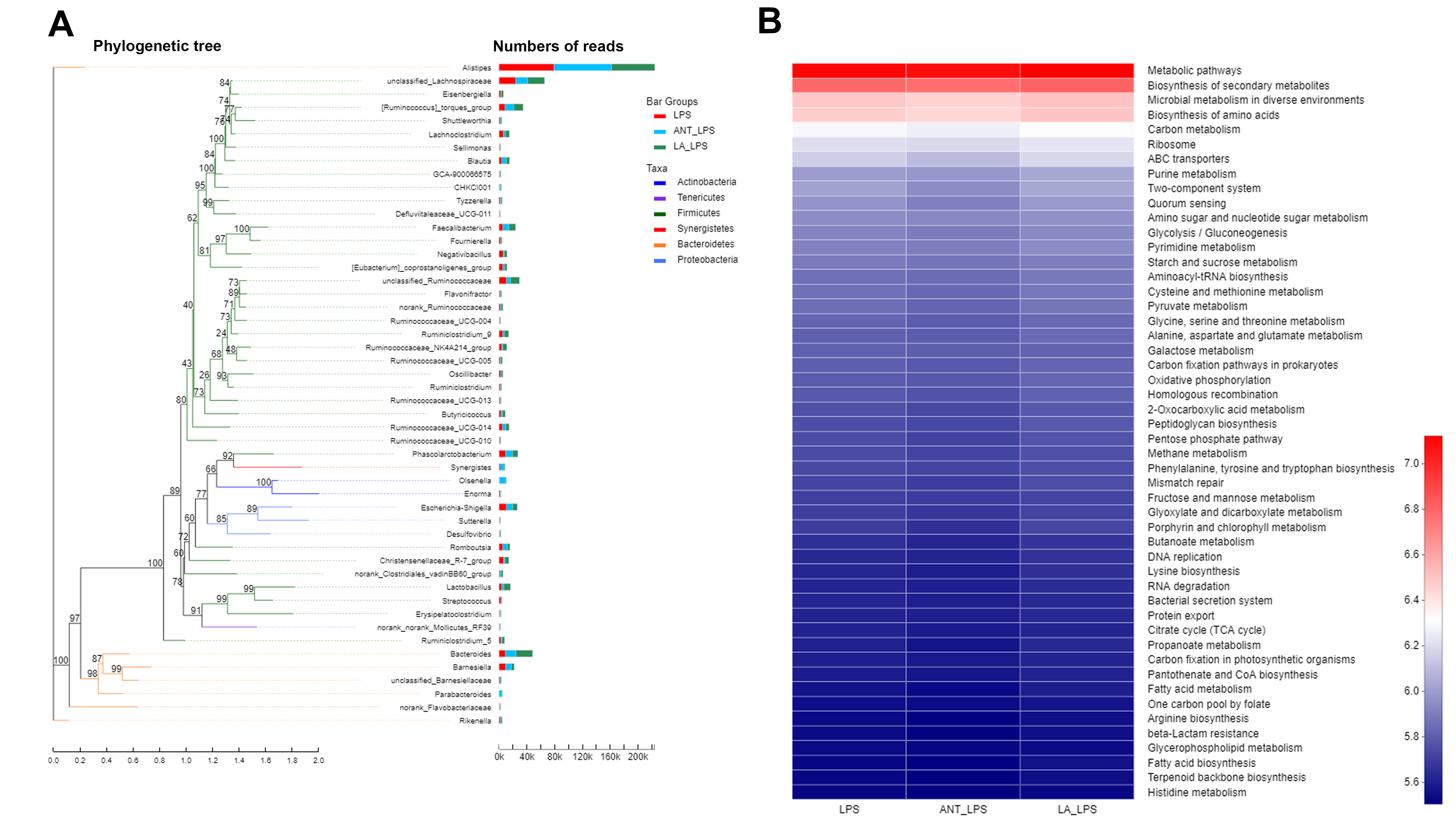

Supplement: Supplementary Figure 1 — (A) Phylogenetic tree of gut microbiota. (B) Heatmap of the predicted functions by PICRUSt analysis. [file Image_1.tif]
